# Supplementary figures and images for: Optimised dissociation and multimodal profiling of prostate cancer stroma reveal fibromuscular cell heterogeneity with clinical correlates
Source: Front Cell Dev Biol. 2025 Nov 25;13:1653780. doi: 10.3389/fcell.2025.1653780 (PMC12685885; doi:10.3389/fcell.2025.1653780)

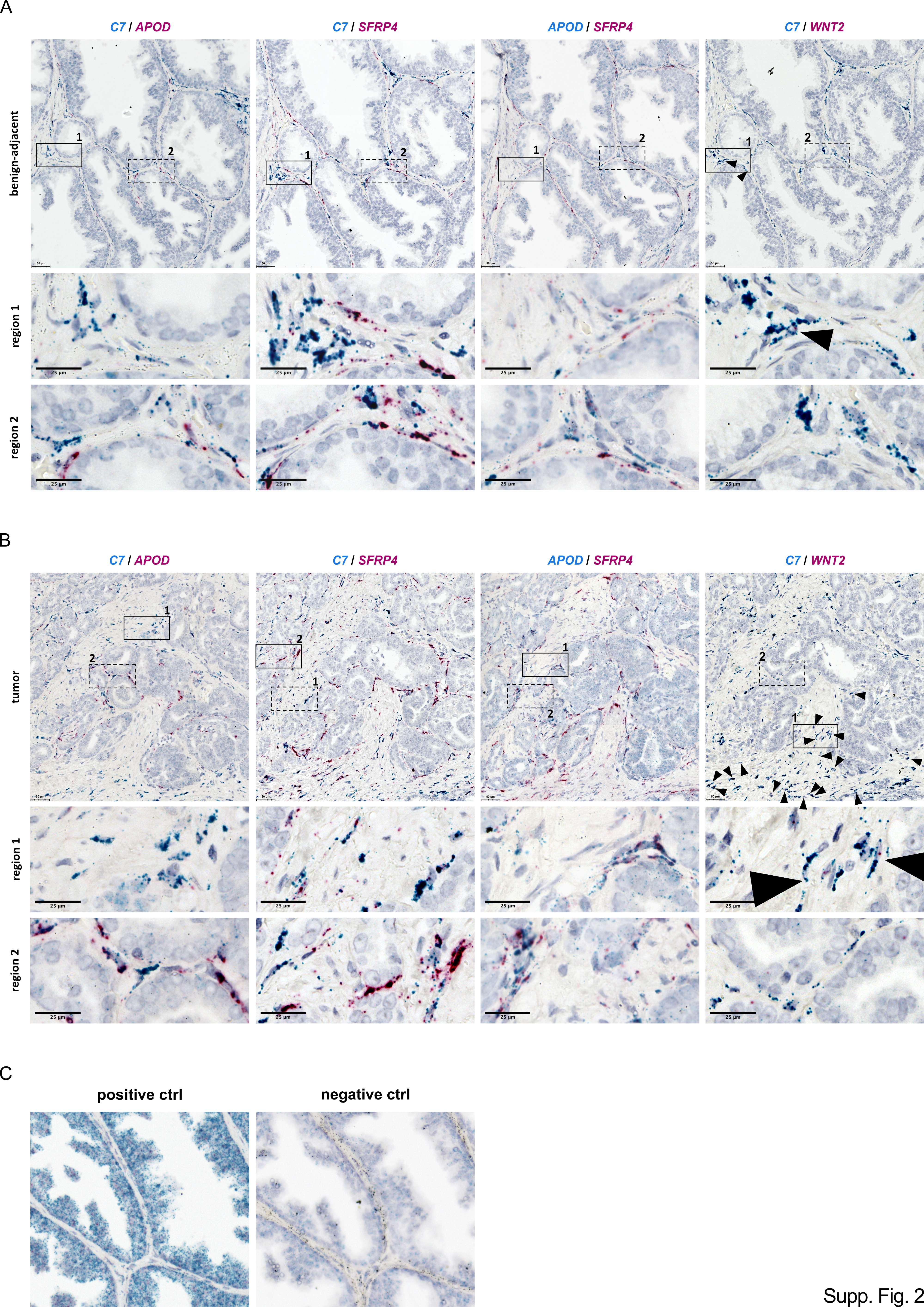

Supplement: Supplementary file 1 [file Image3.tiff]

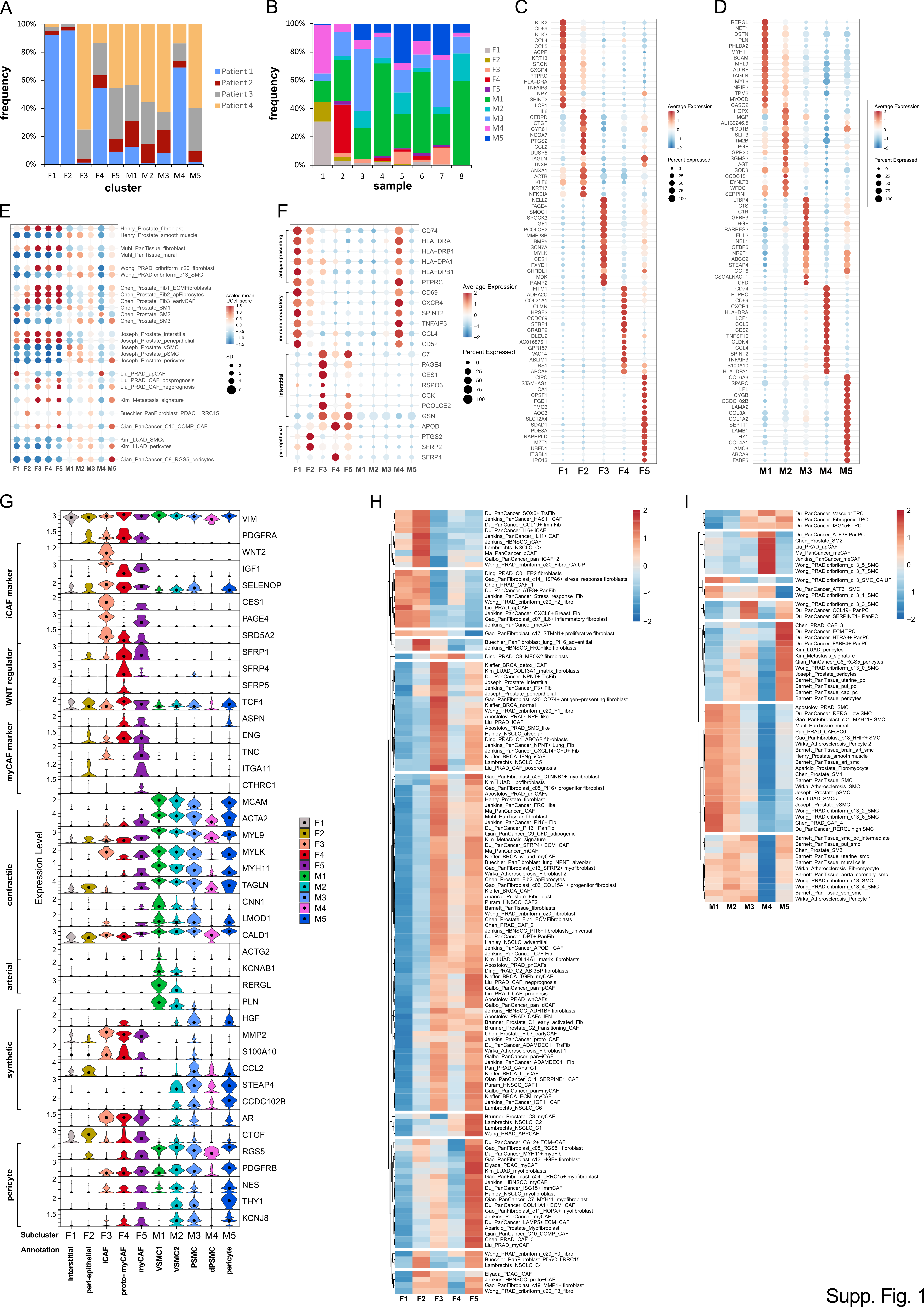

Supplement: Supplementary file 2 [file Image1.tiff]

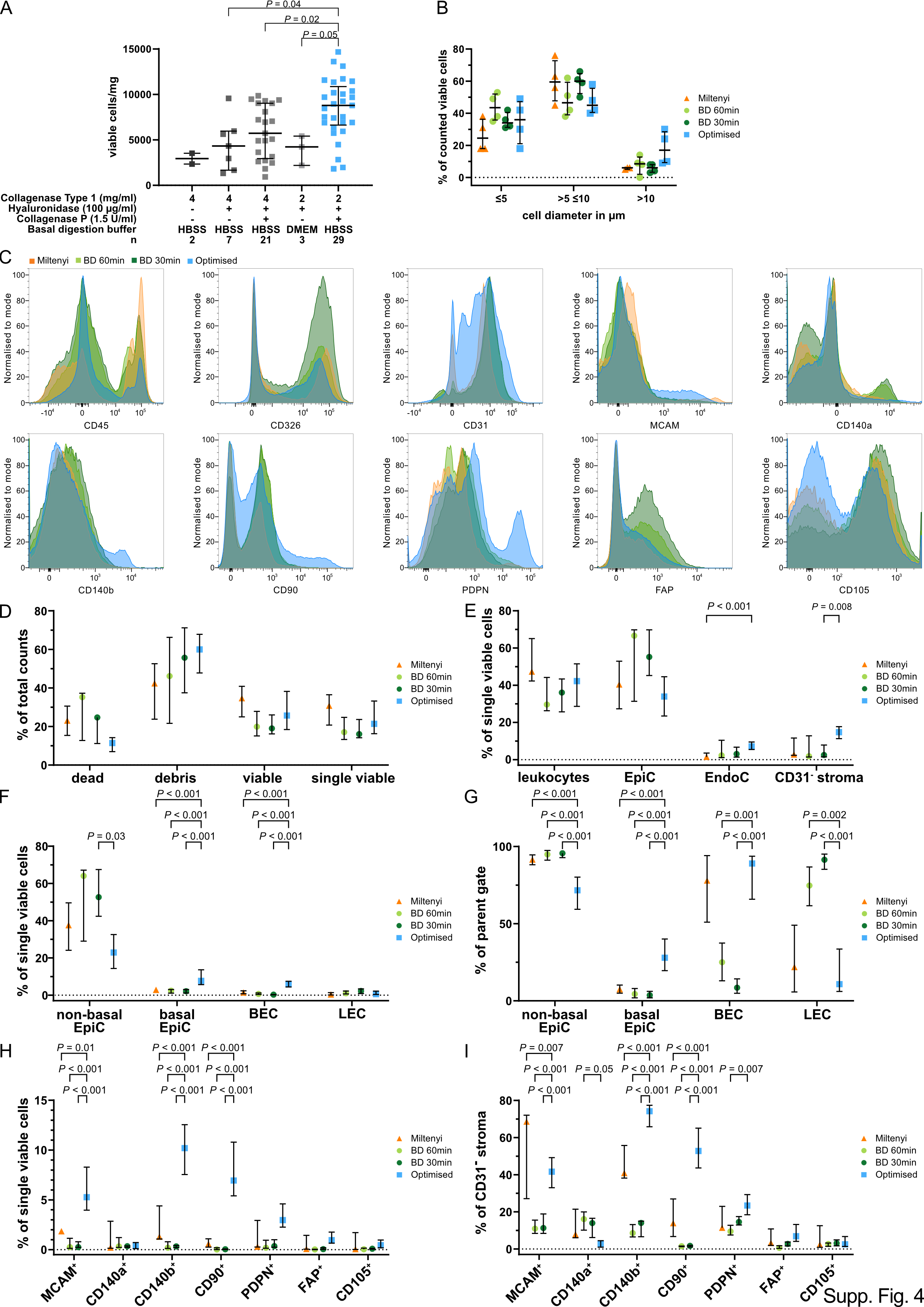

Supplement: Supplementary file 3 [file Image5.tiff]

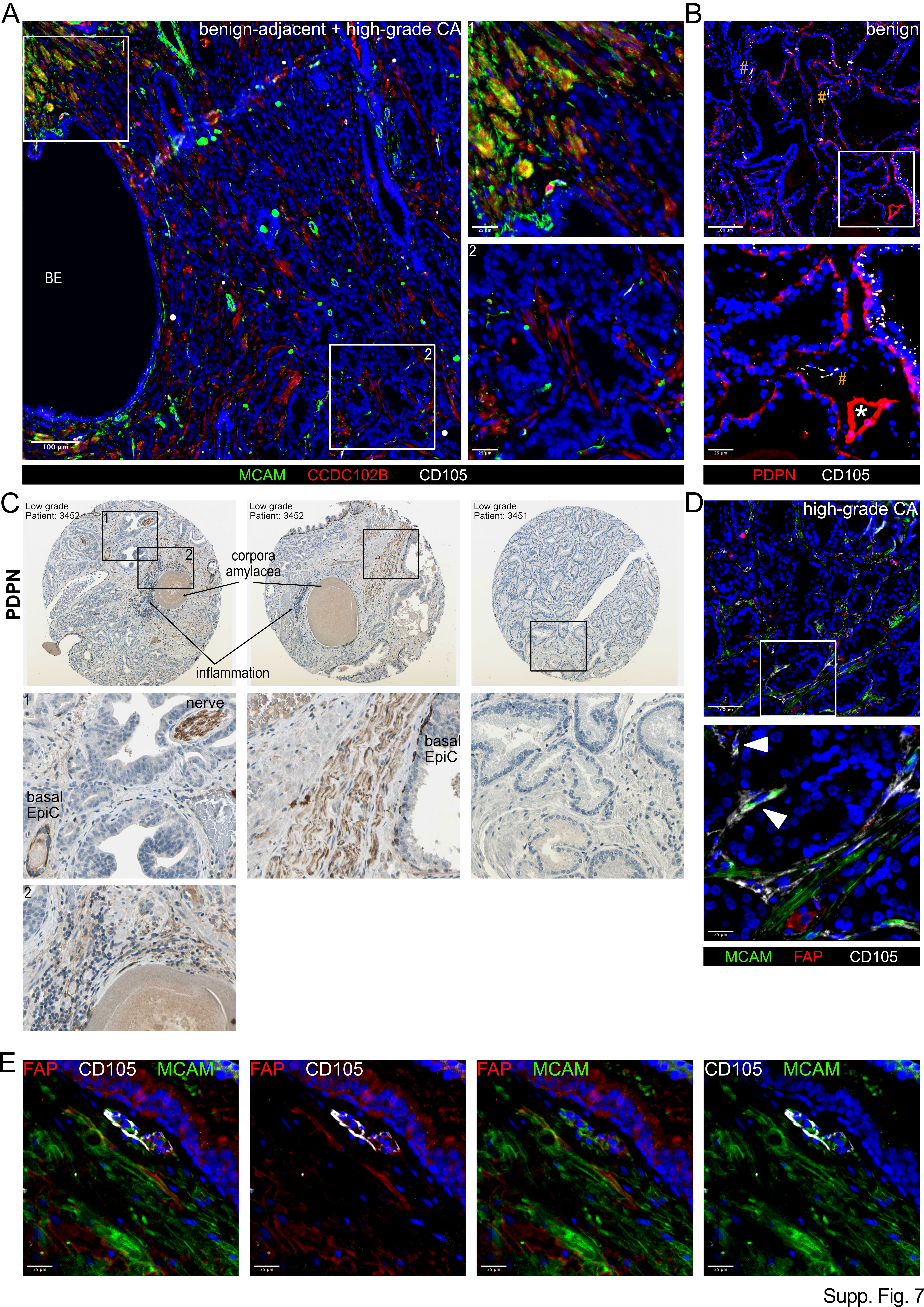

Supplement: Supplementary file 4 [file Image8.tiff]

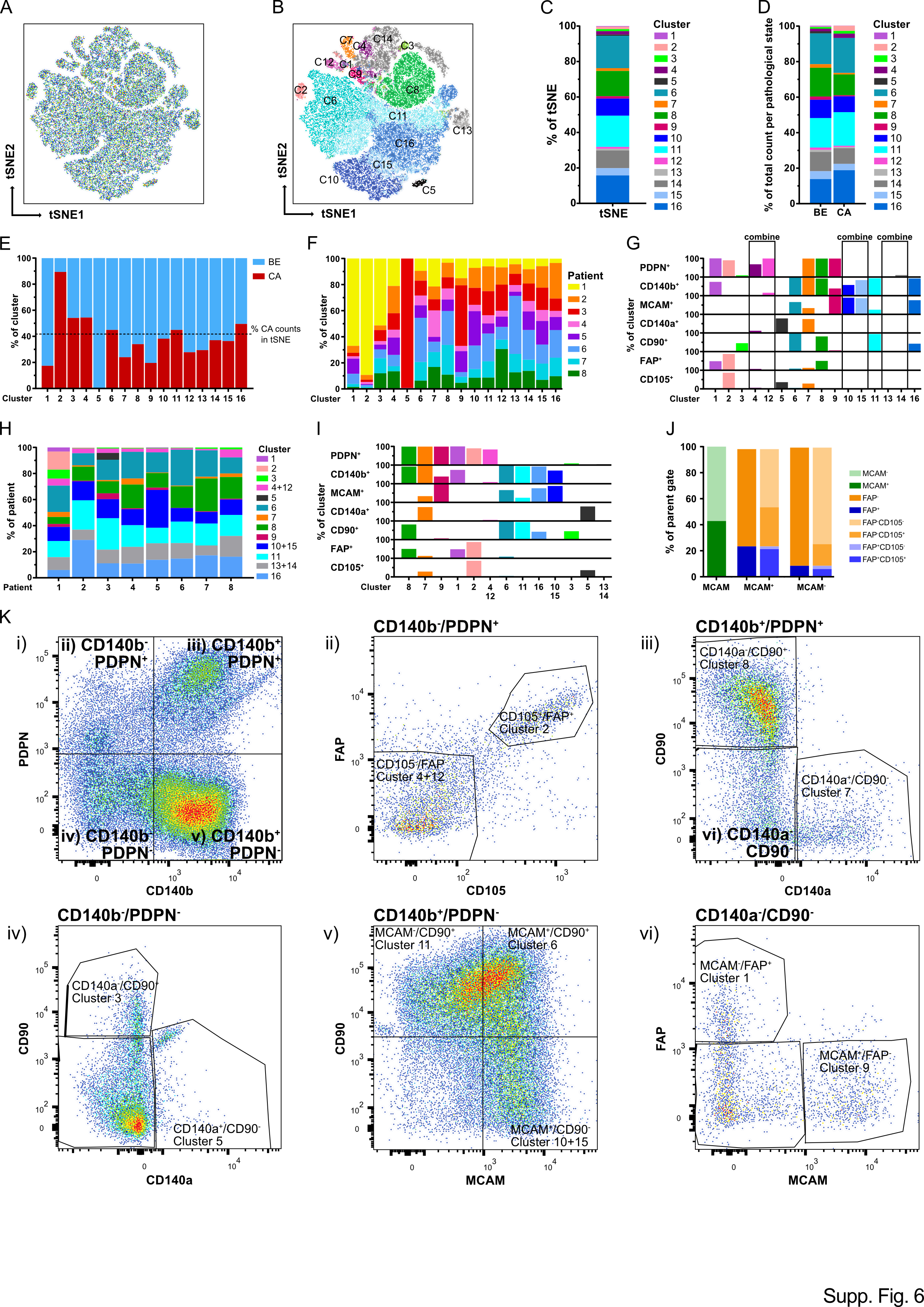

Supplement: Supplementary file 5 [file Image7.tif]

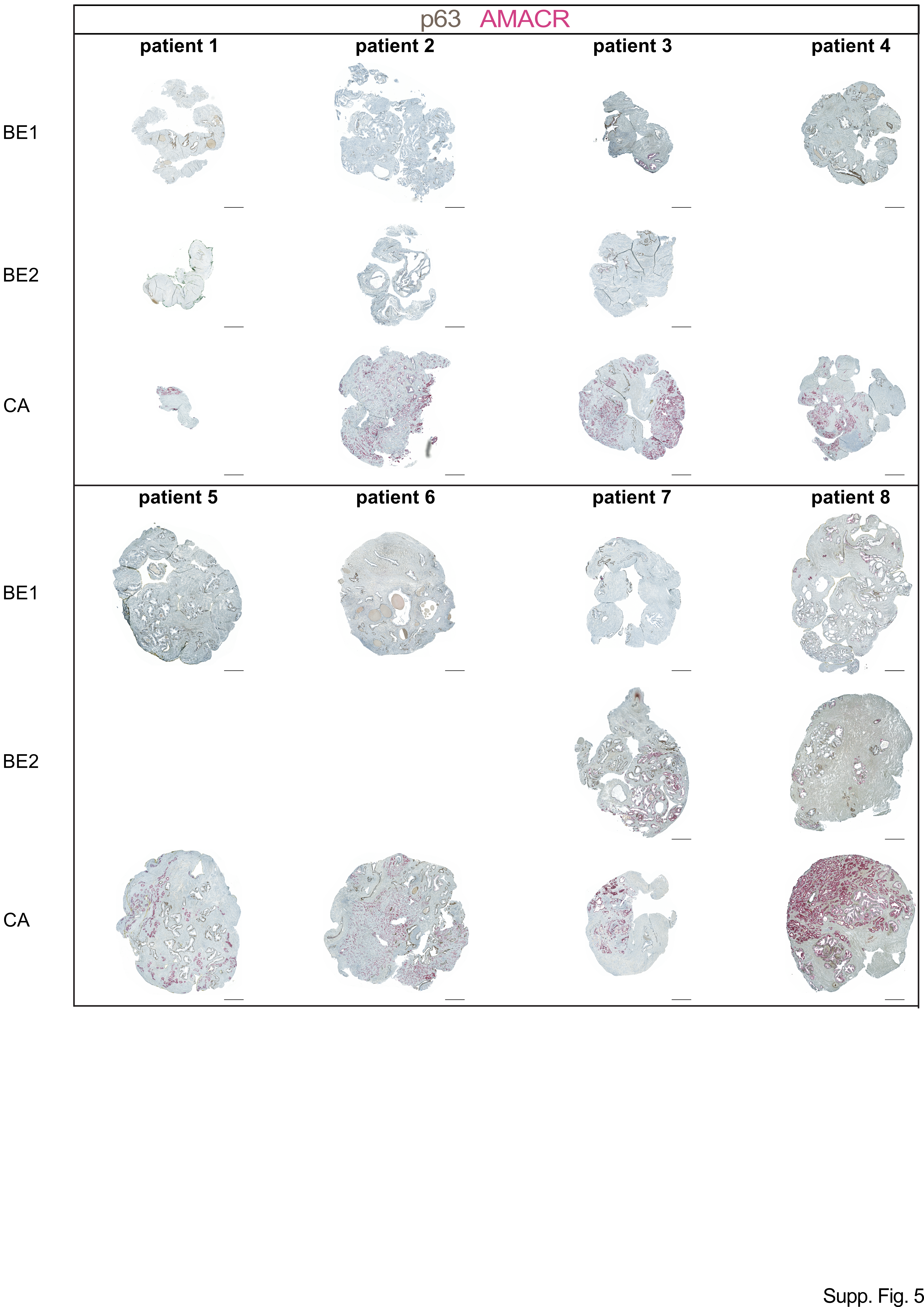

Supplement: Supplementary file 7 [file Image6.tiff]

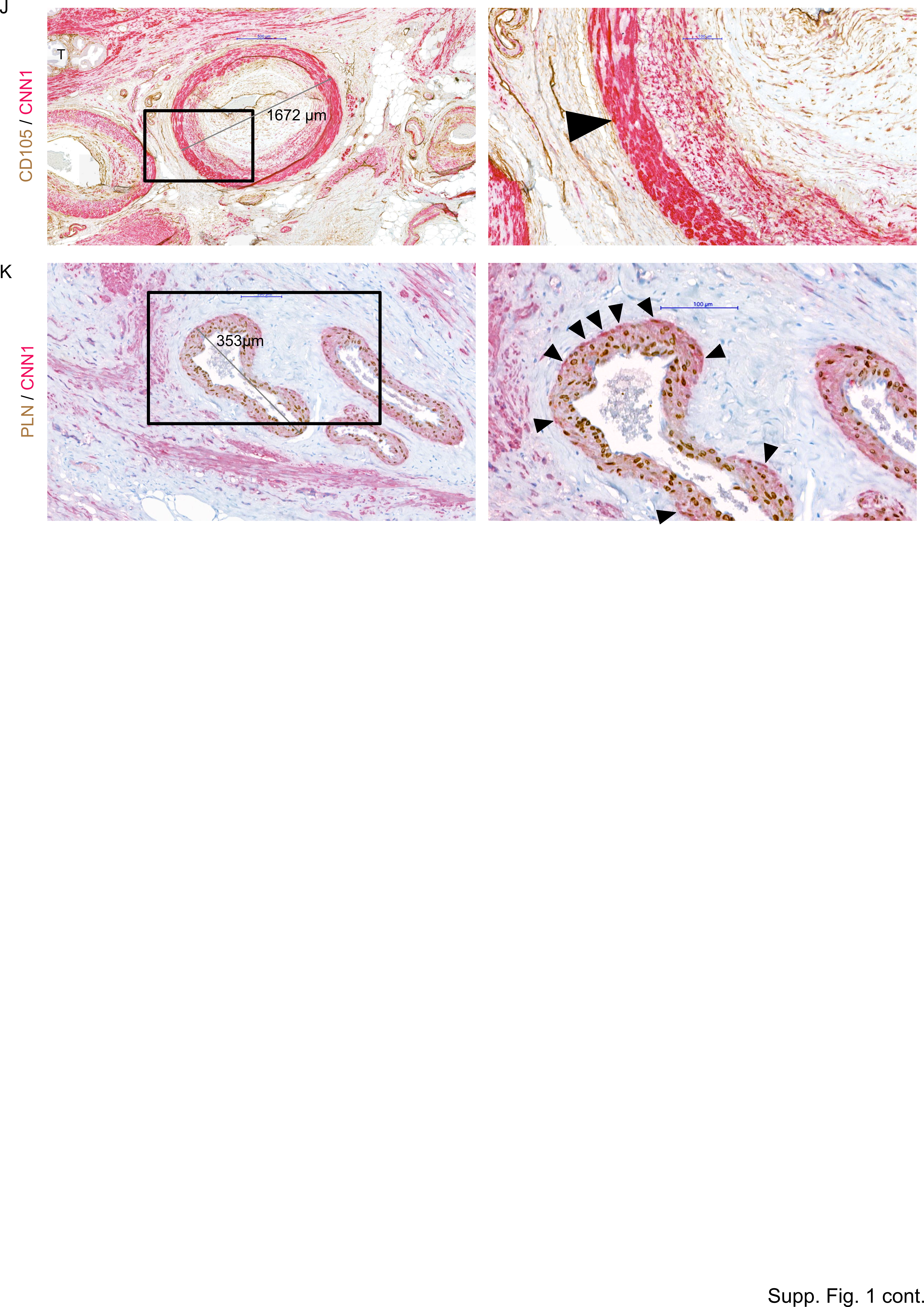

Supplement: Supplementary file 8 [file Image2.tiff]

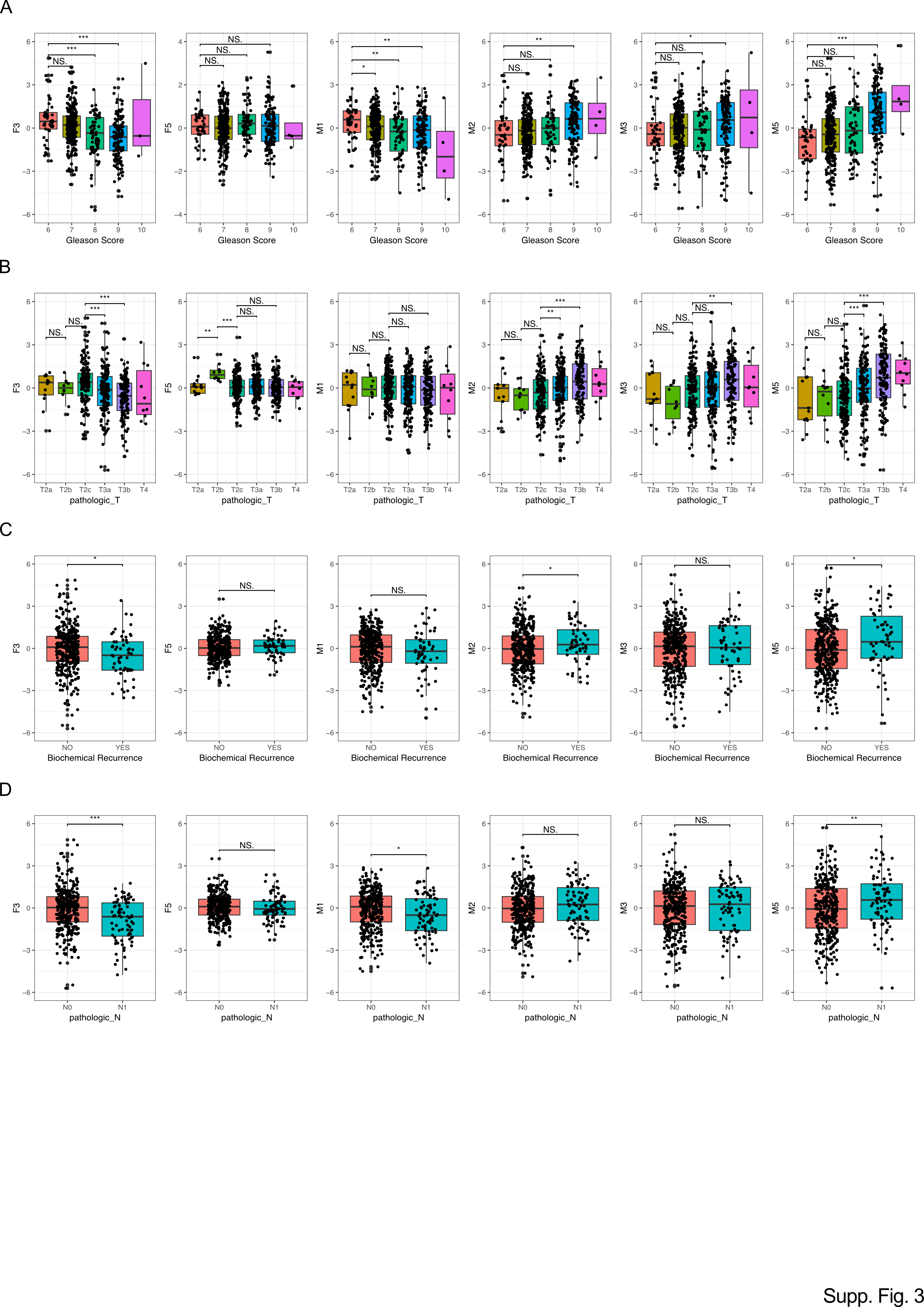

Supplement: Supplementary file 9 [file Image4.tiff]
