# Supplementary material for: Optimised dissociation and multimodal profiling of prostate cancer stroma reveal fibromuscular cell heterogeneity with clinical correlates
Source: Front Cell Dev Biol. 2025 Nov 25;13:1653780. doi: 10.3389/fcell.2025.1653780 (PMC12685885; doi:10.3389/fcell.2025.1653780)
Supplement: Supplementary file 10 [file DataSheet1.docx]

### Supplemental Figure legends

### Supplemental Figure 1 Identification of multiple fibromuscular cell populations *in situ*

**(A-B)** Relative distribution of fibroblast (F) and SMC/mural cell (M) subclusters in re-clustered fibromuscular scRNA-seq data (Heidegger et al., 2022) shown per **(A)** patient and **(B)** sample. **(C-D)** Dotplots showing the average expression of the 15 most significant (P.adj) marker genes for each **(C)** fibroblast and **(D)** SMC/mural cell subpopulation. **(E)** Dotplot depicting the expression of published fibroblast and SMC/mural cell gene signatures across fibromuscular subpopulations. UCell scores were calculated for each gene signature and are represented as a scaled mean per subcluster with dot size inversely proportional to standard deviation (S.D.) to indicate variability within each cluster. **(F)** Dotplot representing average expression of peri-epithelial and interstitial human prostate fibroblast markers (Joseph et al., 2021) across all fibromuscular subpopulations (related to Fig. 1I). **(G)** Violin plot depicting expression levels of selected marker genes demarcating distinct fibroblast (F) and SMC/mural cell (M) subclusters (related to Fig. 1G and 2A). **(H-I)** Heatmaps depicting UCell scores calculated for published gene signatures and scaled per row for **(H)** fibroblast and **(I)** SMC/mural cell subclusters. Rows are clustered by correlation to published signatures using UCell scores represented as the mean of each **(H)** fibroblast or **(I)** SMC/mural cell subcluster. **(J-K)** Dual immunohistochemistry for the indicated antibodies whereby font colour corresponds to staining colour and depicting intense CNN1 staining in the outermost VSMC layer of **(J)** arteries and **(K)** veins (black arrowheads). CD105 expression is observed in adventitial fibroblasts, connective tissue surrounding vessels, endothelial cells and myCAF encircling tumour glands (T). Enlarged images of the boxed regions are shown on the right of the parental image. Source data for panels (E) and (H-I) are provided in the Source Data file.

### Supplemental Figure 2 Duplex-ISH reveals distinct prostate fibroblast populations

**(A-B)** Duplex *in situ* hybridisation of consecutive human benign-adjacent **(A)** and PCa **(B)** tissue sections using the RNA probes indicated, whereby font colour corresponds to staining colour. Sections were counterstained using haematoxylin. Boxed regions are shown enlarged beneath the parental image. Scale bars represent 50 µm (top panels) or 25 µm (middle and lower panels). Images are representative of four independent experiments using tissue sections from three different patients. Black arrowheads denote co-expression of *WNT2* and *C7.* **(C)** Representative images of positive *PPIB* (blue) and *POLR2A* (red) and negative (dapB) control probes run in parallel with experiments shown in (A-B).

### Supplemental Figure 3 Expression of fibromuscular signature genes in TCGA-PRAD cancer and normal prostate tissue cohorts

**(A-D)** Expression levels (combined z-score) of the most significantly upregulated subpopulation-specific genes in the TCGA-PRAD cancer cohort for the indicated fibroblast (F) and SMC/mural cell (M) subpopulations. Boxplots were used to compare **(A)** Gleason score, **(B)** pathological T stage, **(C)** biochemical recurrence and **(D)** N stage. The R package ggsignif (Ahlmann-Eltze and Patil, 2021) was used to perform T-tests. Statistical significance is denoted NS., not significant; *, *P*<0.05; **, *P*<0.01; ***, *P*<0.001. Source data for panels (A-D) are provided in the Source Data file.

### Supplemental Figure 4 Optimisation of tissue dissociation protocols, comparison of cell diameter and flow cytometry analysis

**(A)** Viable cell yield/mg tissue under different dissociation conditions (black and grey boxes) during establishment of the optimised dissociation protocol (blue boxes). Bars represent the median ± interquartile range whereby the number of biological replicates (n) is stated beneath each condition. Statistical significance was determined using Brown-Forsythe and Welch ANOVA tests with Dunnett's T3 correction for multiple comparisons. **(B)** Dissociated cell diameter per protocol calculated via ImageJ (as described in Methods) and stratified according to size as indicated. Bars denote median ± interquartile range of 4 biological replicates per protocol. Statistical significance was determined using Brown-Forsythe and Welch ANOVA tests with Dunnett T3 correction for multiple comparisons. **(C)** Flow cytometry histograms for each marker from a representative experiment illustrating overlap of fluorescence signal distribution across all tissue dissociation protocols. Signal intensity is normalised to mode on the Y-axis to facilitate direct comparison of histogram profiles. **(D-I)** Flow cytometry analysis of each tissue dissociation protocol showing the percentage of **(D)** dead, debris, viable and single viable cells in total counts, **(E)** leukocytes, epithelial cells (EpiC), endothelial cells (EndoC) and CD31^-^ stroma within the single viable cell gate, **(F-G)** EpiC and EndoC further distinguished by PDPN expression into non-basal or basal EpiC, and BEC or LEC as a percentage of **(F)** single viable cells or **(G)** their respective parent gate (EpiC or EndoC), **(H-I)** cells positive for each stromal marker expressed as a percentage of **(H)** single viable cells or **(I)** the CD31- stroma. Bars represent the median ± interquartile range. Statistical significance was determined using (D-G) mixed-effects model with the Geisser-Greenhouse correction and Dunnett correction for multiple comparisons or (H-I) 2-way ANOVA with Dunnett’s correction for multiple comparisons. (D-I) Data are derived from multiple independent experiments using tissue samples from different patients whereby n = 4-6 (Miltenyi), 3-6 (BD 60min and 30 min) or 27-29 (Optimised).

### Supplemental Figure 5 Histopathological staining of eight patient cohort

Dual immunohistochemistry for the basal epithelial cell marker p63 (brown) and tumour cell marker AMACR (red) for histopathological assessment of 4 mm biopsy cores derived from the eight patient-matched cohort. For each patient up to two benign (BE1 or BE2) biopsy cores and one cancerous (CA) biopsy core were sampled. Scale bars represent 500 µm at 20x magnification.

### Supplemental Figure 6 Identification and characterisation of CD31⁻ stromal clusters via tSNE and flow cytometry gating

**(A-B)** tSNE dimensionality reduction analysis of CD31^-^ stroma from flow cytometry data based on the stromal markers MCAM, CD140b, CD90, PDPN, FAP **(A)** without any overlay and **(B)** overlaid with the 16 (unmerged) clusters identified using XShift. **(C-D)** Stacked bar plots depicting the relative distribution of clusters as a percentage of **(C)** the tSNE or **(D)** sample counts according to benign (BE) vs. cancer (CA) histopathology status of the corresponding tissue core. **(E-F)** Relative distribution of **(E)** BE and CA sample counts with overall BE and CA distribution marked as a dotted line and **(F)** patient samples across the 16 clusters. **(G)** Expression of stromal surface markers across the 16 clusters. “Combine” denotes clusters that were subsequently merged into one cluster due to their similar marker expression profiles. **(H)** Relative distribution of the 13 combined clusters across the eight patients. **(I)** Expression of the seven stromal surface markers across the 13 combined clusters. **(J)** Stacked bar plot showing the hierarchical marker distribution within FC-gated cluster 7. The first bar indicates the proportion of MCAM⁺ and MCAM⁻ cells within the cluster. Subsequent bars show the distribution of FAP⁺ and FAP⁻ populations within each MCAM subset, followed by the relative expression of CD105 within the FAP⁺ and FAP⁻ fractions. **(K)** Gating strategy used to identify gated clusters from CD31^-^ stromal flow cytometry data whereby **(i)** gating for the stromal markers CD140b and PDPN results in **(ii)** a CD140b⁻/PDPN⁺ population, which further subdivided into CD105⁺/FAP⁺ (Cluster 2) and CD105⁻/FAP⁻ (Clusters 4+12), **(iii)** a CD140b⁺/PDPN⁺ population, which includes CD140a⁻/CD90⁺ (Cluster 8), CD140a⁺/CD90⁻ (Cluster 7) and CD140a⁻/CD90⁻ (gated in vi), **(iv)** a CD140b⁻/PDPN⁻ population, which further subdivided into CD140a⁻/CD90⁺ (Cluster 3) and CD140a⁺/CD90⁻ (Cluster 5), **(v)** a CD140b⁺/PDPN⁻ population, which includes MCAM⁻/CD90⁺ (Cluster 11), MCAM⁺/CD90⁺ (Cluster 6), and MCAM⁺/CD90⁻ (Clusters 10+15) and **(vi)** the CD140a⁻/CD90⁻ population from (iii), which further separated into MCAM⁻/FAP⁺ (Cluster 1) and MCAM⁺/FAP⁻ (Cluster 9). The gates were set based on FMO and unstained controls.

**Supplemental Figure 7 Distribution of PDPN immunostaining in the benign and malignant prostate**

**(A-B, D-E)** Immunofluorescent staining of prostate tissue sections of indicated pathology using the antibodies stated whereby font colour denotes pseudocolouring in the merged images. Boxed regions are shown to the right **(A)** or beneath **(B, D)** the parental image. Images are representative of at least three independent experiments using tissues derived from at least five patients. **(A)** Immunostaining of the PSMC and mural cell markers CCDC102B and MCAM reveal strong membrane MCAM immunopositivity of PSMC in benign-adjacent regions (BE, marks benign gland) but a considerable decrease within the tumour core, which still contains CCDC102B^+^ PSMC. **(B)** Immunofluorescent staining of PDPN (red) and CD105 (white) in benign prostate tissue confirming PDPN expression by basal prostate epithelial cells that form a single, continuous layer at the basolateral surface of benign glands. Blood vessels with CD105^+^PDPN^-^ vascular endothelial cells (orange hashtags) are readily distinguished from CD105^-^PDPN^+^ lymphatic endothelial cells (white asterisks). **(C)** Images from the Human Protein Atlas (version 24.0) of immunohistochemical staining of prostate tissues exhibiting low-grade PCa using the anti-PDPN antibody CAB008376 (<https://www.proteinatlas.org/ENSG00000162493-PDPN/cancer/prostate+cancer#img>). Boxed regions are shown enlarged beneath the parental image. **(D)** Immunofluorescent staining highlighting heterogeneous non-vessel-associated co-expression of MCAM and CD105 (white arrowheads) potentially indicative of pericyte-/SMC-derived myCAF. **(E)** Immunofluorescent staining of a benign-adjacent prostate tissue section exhibiting signs of activation (as indicated by FAP^+^ epithelial cells and PSMC) highlighting vessel-associated co-expression of MCAM, FAP and CD105 in pericytes.
